# Income and economic exclusion: do they measure the same concept?

**DOI:** 10.1186/1475-9276-11-4

**Published:** 2012-01-27

**Authors:** Emilie Renahy, Beatriz Alvarado-Llano, Maria Koh, Amélie Quesnel-Vallée

**Affiliations:** 1 McGill University, Department of Epidemiology, Biostatistics, and Occupational Health, International Research Infrastructure on Social inequalities in health (IRIS), Peterson Hall, Room 328, 3460 McTavish Street, Montreal, QC H3A 1X9, Canada; 2 Queens University, Department of Epidemiology and Community Health, Carruthers Hall Office 205, 62 Fifth Field Company Lane, Kingston, ON, K7L 3N6, Canada; 3 McGill University, Department of Sociology, Leacock Building, Room 712, 855 Sherbrooke Street West, Montreal, Quebec H3A 2T7, Canada

**Keywords:** Income, economic exclusion, economic hardship, material deprivation, self-rated health, health inequalities

## Abstract

**Introduction:**

In this paper, we create an index of economic exclusion based on validated questionnaires of economic hardship and material deprivation, and examine its association with health in Canada. The main study objective is to determine the extent to which income and this index of economic exclusion index are overlapping measurements of the same concept.

**Methods:**

We used the Canadian Household Panel Survey Pilot and performed multilevel analysis using a sample of 1588 individuals aged 25 to 64, nested within 975 households.

**Results:**

While economic exclusion is inversely correlated with both individual and household income, these are not perfectly overlapping constructs. Indeed, not only these indicators weakly correlated, but they also point to slightly different sociodemographic groups at risk of low income and economic exclusion. Furthermore, the respective associations with health are of comparable magnitude, but when these income and economic exclusion indicators are included together in the same model, they point to independent and cumulative, not redundant effects.

**Conclusions:**

We explicitly distinguish, both conceptually and empirically, between income and economic exclusion, one of the main dimensions of social exclusion. Our results suggest that the economic exclusion index we use measures additional aspects of material deprivation that are not captured by income, such as the effective hardship or level of economic 'well-being'.

## Introduction

In most developed countries, glaring health inequalities exist that reflect, but are not reducible to, lifestyle and health behaviours, and that bear a strong relationship with socioeconomic position [1-3]. This situation has been deemed of such concern to researchers and policymakers alike that limiting these inequalities has recently been put at the forefront of the policy agenda by the World Health Organisation [4]. In Canada for instance, the Government of Quebec passed in 2002 *An Act to combat poverty and social exclusion *(R.S.Q., c. L-7). This was the first legislation of its kind to be passed in North America and it was received positively by the public health community [5]. Starting in 2008, many other Canadian provinces introduced poverty reduction strategies or action plans [6]. There will be a need for monitoring and evaluating the impact of such policies and strategies on health. Acknowledging the existence of a social gradient in health (that goes beyond the dichotomy of the richest and the poorest), we deemed it important to assess different measures of economic position.

Socioeconomic position is a multidimensional concept, most commonly operationalised in health inequalities research by education, income, and employment-based social class or social status [7]. While some early research attempted to establish which of these indicators was the most stable or strongest predictor of health, the consensus is now that, although correlated, these measures are not interchangeable [8,9]. Indeed, it appears that these indicators do not capture the same constructs of material resources, power and prestige [8].

Moreover, each of these dimensions of socioeconomic position has been subjected to studies of the internal consistency of their various indicators. For instance, material resources have been captured through numerous indicators, of which income is only one facet, which may not accurately capture the extent of material deprivation in the population. In this paper, we examine the extent to which a direct, "gold-standard" measure of income overlaps with other measures of economic deprivation or economic exclusion in Canada.

## Background and objectives

Limitations to income measurement are rarely thoughtfully acknowledged in health research [8]. First, income can be measured in various ways (e.g. through self-report or tax file linkage; using individual or household measures; pre or post-tax; as main source of income, or through all possible sources of income; not to mention all possible transformations and use of percentiles). This contributes to study findings heterogeneity, as different measurement can introduce unmeasured confounding: in a dose-response perspective, decreases in income should lead to poorer health, but if income is measured as individual income from salary, the relationship may be weakened by the fact that household income and wealth can mitigate this negative impact. Furthermore, income is typically a sensitive question in surveys, which leads to high levels of missing cases that are not randomly distributed in the population, and thus often biased at the extremes of the distribution [10]. Finally, and perhaps even more importantly, income might not accurately reflect the material conditions of individuals and households. Indeed, the provision of in-kind social services such as housing assistance and food stamps cannot be captured through income measures [11,12].

Alternative indicators attempt to more explicitly measure the effective economic hardship or the level of 'well-being' instead of implicitly assuming it from income. For instance, economic hardship and material deprivation [13] are conceptually more proximal measures of effects of poverty than income. Economic hardship measures mainly focuses on the extent of deprivation in possessing goods, accessing services and engaging in certain activities [14,15]. Indicators of economic hardship include cutbacks on monthly expenditures, inability to pay bills, difficulty in meeting basic needs such as food and shelter. Material deprivation measures are assessing the inability to afford some basic goods, lifestyle or opportunities "to participate in a way identified generally as appropriate in [a given] community" [13]. Interestingly, while these indicators are undoubtedly conceptually linked to income, a number of previous studies have shown that these constructs do not overlap perfectly [16-19].

Moreover, while there is a wealth of research linking income to health, studies linking material deprivation [20-25] and economic hardship [26-31] to health are scant, particularly in the Canadian context.

Thus, in this study, we take advantage of the very rich information and innovative measurement of the Canadian Household Panel Survey - Pilot (CHPS-Pilot) [32] to create an index of economic exclusion based on validated questionnaires of economic hardship and material deprivation, and examine its association with health in Canada.

More specifically, our main study objective will be to determine the extent to which income and an economic exclusion index are overlapping measurements of the same concept. We will pursue this objective by answering the following two research questions:

1. Are the same population groups at risk identified when using income and the index of economic exclusion?

2. Are both measures, income and the index, independently associated with the risk of poor self-rated health?

## Methods

### Sample

The CHPS-pilot is a cross-sectional survey initiated by Statistics Canada. The overall objective was to develop a longitudinal household panel survey to monitor the evolution of key indicators on work, health, education and family deemed to be salient for both the well-being of Canadians and for the development of social policies. The pilot study took place in the fall 2008 in Canada. The household survey design entails that all individuals in a household are interviewed (and eventually followed); for the pilot study a total of 3, 181 individuals 15 years and over and nested within 1, 627 households were interviewed. As this non-random sample drew respondents from only four out of ten provinces (Ontario, Quebec, New Brunswick and Saskatchewan), it cannot be seen as representative of the Canadian population.

**Figure 1:**
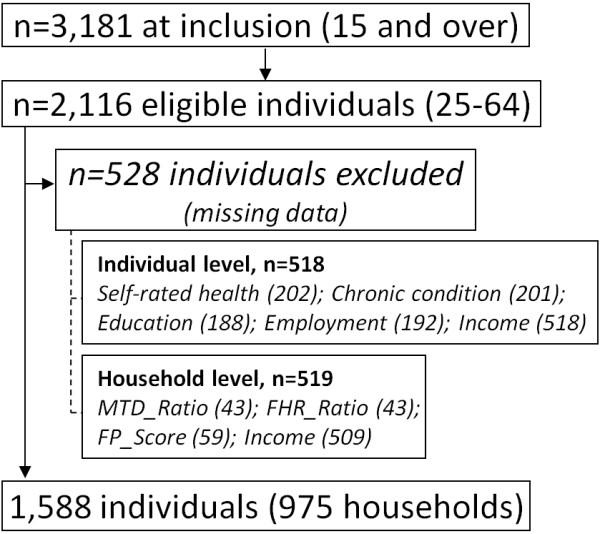
**CHPS-Pilot 2008, sample selection**.

Figure 1 shows the sample restrictions imposed in our analyses. First, we considered only respondents aged from 25 to 64 years. Second, 528 individuals were not included in these analyses because of missing information on key variables; 518 individuals had information missing on an individual characteristic (socioeconomic status, health status, individual income) and 519 on at least one household variable (269 households missing either household income or at least one of the three dimension of the economic exclusion index). Finally, as we created the household income as the sum of individual income of all household members (because we wanted a consistent sample across our household and individual income analyses, see below), we had to delete not only individuals with missing data on their own income, but also the complete household. This unfortunately resulted in the loss of 263 households and 509 individuals. In all, we considered 1, 588 individuals aged 25 to 64 without missing information, nested within 975 households.

### Creation of the index of economic exclusion

We created an index of economic exclusion measured at the household level. As a household panel survey, the CHPS-Pilot featured two questionnaires: one individual questionnaire, filled out by each member of the household; one household questionnaire filled out by the individual member of the household designated as most knowledgeable. We therefore imputed the values of this household-level index to all member of the same household.

We used three sets of questions that capture most of the sub-dimensions of economic exclusion found in the literature:

1) Material deprivation (MTD)

2) Food, housing and financial risk or economic hardship (FHR)

3) Financial products or wealth (FP)

For all these dimensions, greater values indicate higher levels of deprivation or financial risk.

First, the MTD dimension consisted of a list of 10 usual material necessities, as outlined in Table 1. This dimension explicitly measures whether the household is able to afford certain material resources. If the primary respondent stated that the household did not possess the item or was not engaged in the activity, he/she was then further prompted to state whether this was because the household could not afford it. Different ways of aggregating these responses have been found in the literature [33-37]. Moreover, international studies assess material deprivation using a different number of items and different wording for response categories [38]. We therefore decided to create a new synthetic variable to allow comparability with further studies, by calculating the ratio of the number of deprivation experiences divided by the total number of items answered, such that a higher value (on the theoretical range from 0 to 1) indicates a higher level of material deprivation.

**Table 1:** Material deprivation*

1.	Do you save regularly at least about $20 per month for rainy days or for retirement?
2.	Do you have fresh fruits and vegetables every day?

3.	Do you have a small amount of money to spend each week on yourself?

4.	Do you have meat, fish or vegetarian equivalent every other day?

5.	Are you able to replace worn out furniture?

6.	Do you have appropriate clothes for job interviews?

7.	Are you able to get around either by having a car or by using a monthly bus, subway, or commuter train pass (or equivalent)?

8.	Are you able to have friends or family over for a meal at least once per month?

9.	Do you have at least two pairs of shoes, including one to wear outside in the winter?

10.	Are you able to buy modest presents for family or friends at least once per year?

*Do you have/Are you able to... If no, is it because you cannot afford it?

We performed the same ratio calculation for the FHR dimension, which was based on 8 questions presented in Table 2. Here, questions assessed the inability to pay bills or the experience of having difficulties making ends meet. This questionnaire therefore aims at measure the effective monetary hardship. Here too, a greater value indicates a higher level of risk.

**Table 2:** Food, household and financial risks

1.	In the last 12 months, have you ever had to eat less because you did not have enough money to buy food?
2.	In the last 12 months, have you ever served food that you thought was not good for you because you did not have enough money to buy good quality food?

3.	In the last 12 months, did you ever miss paying an electricity, gas or utility bill on time because you were short of money?

4.	In the last 12 months, did you ever miss paying the rent or mortgage on time because you were short of money?

5.	In the last 12 months, did you ever pawn or sell something because you were short of money?

6.	In the last 12 months, did you ever ask for financial help from friends or family because you were short of money?

7.	In the last 12 months, did you ever use a food bank?

8.	In the last 12 months, did you ever ask for help from welfare or community organizations because you were short of money?

Finally, we created a score to assess the FP dimension through ownership status and ability to face unexpected expenditures, where higher values indicate greater levels of deprivation of this potential. This dimension aims at characterize the wealth of the household or its access to financial products. We created a variable with three categories for home ownership status: own without mortgage (0), own with mortgage (1), rent (2). The scale reflects the fact that 1. those who own without a mortgage have built more equity and are thus potentially wealthier (this is an assumption, as the value of the property was not available in the survey); and 2. that owning one's residence is still better than renting, even with a mortgage (which may lead to higher monthly payments than the rent), because it offers that equity and wealth-building potential (a process sometimes referred to as "forced savings"). We then considered the capacity to face unexpected expenditures (e.g. using savings, credit, sell an asset, borrow from relatives - Yes = 0, Not be able to face these expenditures = 1) through a multiple choice question (see Table 3). This decision rule is probably conservative, as the various options offered are not equivalent with regards to the financial risk they incur (i.e. some of these options may lead respondents into greater financial debt). However, we decided to give them the same weight, as we cannot assess the full implications of the financial choices made by respondents (if for instance, the market is depressed and the value of savings low, it may be a sound financial decision to take advantage of low interest rates rather than turn unrealized losses into realized losses). We finally summed these two variables which reflect the fact that, the greater the score of FP (range from 0 to 3), the least access to financial resources. We did not include 33 households and their respective members in our analysis because of missing information.

**Table 3:** Ability to face to unexpected expenditures. If you had to make an unexpected expenditure today of $5, 000 or more, would you...?

1.	Use savings
2.	Borrow from a friend or relative

3.	Use credit cards

4.	Use a line of credit

5.	Arrange for a loan

6.	Sell an asset

7.	Not be able to handle this unexpected expenditure

**Figure 2:**
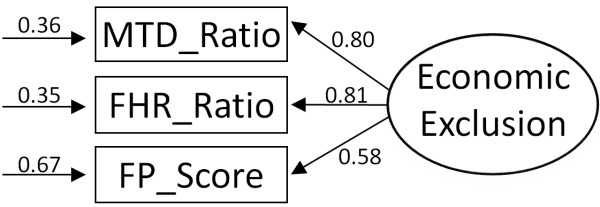
**Confirmatory factor analysis**.

We finally used confirmatory factor analysis based on structural equation modeling to reduce the three economic dimensions (measured through MTD, FHR and FP) into one underlying latent construct or factor of economic exclusion (see Figure 2). The observed dimensions introduced into the CALIS Procedure (SAS^®^) are those previously defined (MTD- and FHR-ratios, FP-score). We defined the estimation of error terms as free and each factor variances fixed at 1. Our data meet the basic criteria of sample size (5-20 cases per parameter estimate). We finally created a factor score for each household using the SCORE Procedure (SAS^®^) and imputed this value to every member of the household.

### Income

We used a log transformation (log(inc/10000+1)) of annual individual gross income. About 70% of income data came directly from a linkage with income tax data from the Canadian Revenue Agency. For the other respondents, the information was either recorded through the CHPS-Pilot questionnaire (detailing amounts by source of income), or missing. While it would have been useful to use net income (after taxes and transfers) to more accurately assess disposable income, this indicator was unfortunately not made available. We created the household annual income before tax by summing the individual incomes of all members of the household aged 15 or more. We imputed a value of 0 to individual income to those individuals under 25 years old, living with their parents and not employed (n = 85). We were not able to create this total household income when at least one member of the household was missing data for his/her own income.

### Socio-demographic and health variables

We used the following social and demographic covariates: sex, age, marital status (single, coupled, divorced or separated, widowed), and immigration status (immigrant/non-immigrant). The respondents' socioeconomic status was characterized by level of education (less than high school, high school or more than high school) and employment status (employed, unemployed or out of the labour force (OLF)). Regarding household level variables, we considered the number of people living in the household as well as the composition of the household: single individual, couple without children, couple with child(ren), single parents and other living arrangements (refers generally to non-coupled cohabitating roommates).

To assess individual health status, we used chronic conditions (1 if the person reported at least one chronic condition from a list of 13 main chronic conditions, 0 otherwise) as well as self-rated health status. We created a dichotomous variable to estimate the probability of being in poor health (fair or poor vs. good, very good or excellent) using logistic regression.

### Statistical analyses

Using the index of economic exclusion, we first performed mean comparisons (or correlations) of the global index of economic exclusion by age, gender, migration status, marital status, education, employment status, and health status. This first step was meant to answer question 1.

Secondly, to answer question 2, we estimated multilevel analyses to assess the association of income (at both the individual - Level 1 - and household levels - Level 2) and the index of economic exclusion (measured at the household level - Level 2) with self-rated health (measured at the individual level - Level 1) controlling for individual and household characteristics. We estimated a set of nested multilevel models using the xtmelogit Stata^® ^procedure. To assess the existence of a household effect, we estimated a model without covariates (M0, empty model). The presence of the household effect was estimated for each model with a median odds ratio (MOR) that quantifies the variation between household clusters; the greater the MOR, the greater the variation [39]. We then successively estimated a model with individual characteristics plus household-level information, among them economic exclusion (M1). We then estimated models without economic exclusion but adding individual income (M2a) or household income (M2b) and finally, models with both the index of economic exclusion and individual (M3a) or household income (M3b).

## Results

In our sample of 1, 588 respondents, there were more women (52.1%) than men, and more non-immigrants (79.0%) than immigrants (Table 4). Almost two third of respondents had an educational level higher than high school (63.1%), were employed (77.1%), and were in a couple relationship (74.0%). Regarding health status, 11.1% rated their health status as fair or poor, while 45.7% reported at least one chronic or long-term condition. The 975 households interviewed were composed of single individuals (23.1%), couples without children (27.2%), couples with children (35.2%), and single parents (11.3%).

**Table 4:** CHPS-Pilot - Sample characteristics (1, 588 individuals nested within 975 households)

	n(%)		n(%)
***Individual level***	***(ni = 1, 588)***		

**Age**		**Self-rated health**	
*25-34*	337 (21.2%)	*Poor*	176 (11.1%)
*35-44*	431 (27.1%)	*Good*	1412 (88.9%)
*45-54*	432 (27.2%)	**Chronic condition**	
*55-64*	388 (24.4%)	*Yes*	726 (45.7%)
**Gender**		*No*	862 (54.3%)
*Male*	761 (47.9%)	**Education**	
*Female*	827 (52.1%)	*Less than high school*	226 (14.2%)
**Immigration status**		*High school*	361 (22.7%)
*Immigrant*	334 (21.0%)	*More than high school*	1001 (63.1%)
*Non immigrant*	1254 (79.0%)	**Employment**	
**Marital Status**		*Employed*	1224 (77.1%)
*Single*	222 (14.0%)	*Unemployed*	59 (03.7%)
*Couple*	1175 (74.0%)	*OLF*	305 (19.2%)
*Divorced/Separated*	165 (10.4%)		**μ (σ)**
*Widowed*	26 (01.6%)	**Individual Income**	1.420 (0.675)

***Household level***	***(nh = 975)***		**μ (σ)**

**Composition**		**Household Income**	1.869 (0.706)
*Single*	225 (23.1%)	**MTD_Ratio**	0.082 (0.165)
*Couple without child*	265 (27.2%)	**FHR_Ratio**	0.081 (0.169)
*Couple with children*	343 (35.2%)	**FP_Score**	1.124 (0.961)
*Single parents*	110 (11.3%)	**Factor of Exclusion**	0 (0.940)
*Other*	32 (03.3%)		

The means of the three indicators used to create the index were 0.082 (0.165) for the MTD_ratio, 0.081 (0.169) for the FHR_ratio and 1.124 (0.961) for the FP_score. Correlations between these three indicators vary from 0.48 to 0.65 (Table 5). By construction, the mean of the index of economic exclusion is 0, and thus, the higher the index of economic exclusion, the higher the exclusion (positive values) while the lower the index, the lower the exclusion (negative values). Finally, the mean individual and household log-incomes were respectively 1.420 (0.675) and 1.973 (0.696).

**Table 5:** Means Pearson correlation coefficients of income and economic dimensions with economic exclusion.

	Economic Exclusion	MTD_Ratio	FHR_Ratio	FP_Score	Alpha
***Individual level***	***(ni = 1, 588)***				

Individual income	-0.361***				

***Household level***	***(nh = 975)***				

MTD_Ratio		1			0, 647
FHR_Ratio		0.650***	1		0, 666
FP_Score		0.493***	0.4786***	1	0, 787
Household income	-0.497***				

*** p < .0001

As expected, we see that economic exclusion is negatively correlated with both individual and household log-income. While the correlation between economic exclusion and income is relatively low (under 0.4), it was significant in both cases, and slightly stronger for household income, which is conceptually congruent (given that the index was also measured as a household characteristic). These correlation coefficients provide the first indication that income and a more explicit measurement of economic exclusion are not perfectly overlapping constructs.

In Table 6 a positive score of economic exclusion indicates a greater exclusion than the mean, while a negative score indicates lower-than-average exclusion. We can see that exclusion decreased with increasing age and education. It was also lower among the employed, and interestingly among those out of the labor force as well, although the unemployed reported higher levels of exclusion. Similarly, lone parents and immigrants reported significantly higher levels of exclusion. Finally, while exclusion was higher among those who reported poor health, the difference was less significant with those who reported chronic condition.

**Table 6:** Mean economic exclusion, individual and household income by categories (n = 1, 588)

	Economic Exclusion		Individual income		Household income	
	**μ (σ)**	**p_value**	**μ (σ)**	**p_value**	**μ (σ)**	**p_value**

***Individual level***						

**Age**		< .0001		< .0001		0, 005
*25-34*	0.169 (0.833)		1.240 (0.636)		1.860 (0.661)	
*35-44*	0.078 (0.860)		1.454 (0.614)		1.972 (0.705)	
*45-54*	-0.041 (0.876)		1.487 (0.726)		2.029 (0.741)	
*55-64*	-0.198 (0.858)		1.463 (0.688)		2.011 (0.656)	
**Gender**		0, 056		< .0001		0, 100
*Male*	-0.079 (0.836)		1.606 (0.634)		2.003 (0.684)	
*Female*	0.037 (0.895)		1.248 (0.668)		1.946 (0.707)	
**Immigration status**		0, 009		< .0001		< .0001
*Immigrant*	0.107 (0.862)		1.229 (0.812)		1.837 (0.879)	
*Non immigrant*	-0.139 (0.866)		1.471 (0.624)		2.010 (0.634)	
**Marital Status**		< .0001		0, 010		< .0001
*Single*	0.311 (1.071)		1.283 (0.537)		1.596 (0.632)	
*Couple*	-0.116 (0.731)		1.446 (0.702)		2.114 (0.663)	
*Divorced/Separated*	0.356 (1.163)		1.404 (0.633)		1.528 (0.654)	
*Widowed*	-0.158 (1.160)		1.513 (0.638)		1.651 (0.548)	
**Education**		< .0001		< .0001		< .0001
*Less than HS*	0.265 (1.065)		1.082 (0.610)		1.611 (0.613)	
*High school*	0.082 (0.960)		1.294 (0.585)		1.919 (0.626)	
*More than HS*	-0.093 (0.763)		1.541 (0.685)		2.074 (0.709)	
**Employment**		< .0001		< .0001		< .0001
*Employed*	-0.098 (0.712)		1.556 (0.613)		2.072 (0.633)	
*Unemployed*	0.352 (1.010)		1.113 (0.712)		1.672 (0.746)	
*OLF*	0.132 (1.239)		0.933 (0.663)		1.635 (0.801)	
**Self-rated health**		< .0001		< .0001		< .0001
*Poor*	0.554 (1.327)		1.076 (0.607)		1.640 (0.665)	
*Good*	-0.007 (0.765)		1.463 (0.671)		2.015 (0.689)	
**Chronic condition**		< .0001		0, 038		0, 167
*Yes*	0.063 (0.999)		1.381 (0.680)		1.947 (0.697)	
*No*	-0.058 (0.736)		1.452 (0.669)		1.996 (0.696)	

***Household level***						

**Composition**		< .0001		0, 083		< .0001
*Single*	0.291 (1.1563)		1.433 (0.616)		1.433 (0.616)	
*Couple without child*	-0.202 (0.736)		1.412 (0.652)		2.060 (0.596)	
*Couple with children*	-0.049 (0.728)		1.485 (0.668)		2.157 (0.683)	
*Single parents*	0.623 (1.100)		1.312 (0.574)		1.444 (0.586)	
*Other*	0.053 (1.070)		1.279 (0.566)		1.733 (0.513)	

As mentioned above, the index of economic exclusion we have created was inversely associated with income (either individual or household). However, the negative correlation was low and we can observe some additional distinguishing features. First, while log-income increased with age until age 55 (it then decreased), exclusion 'continuously' decreased with age. Moreover, while males have higher income than women, there was no significant difference regarding economic exclusion. Finally, those with chronic conditions earned significantly less money than those without chronic conditions, although they did not report more economic exclusion.

Nested multilevel logistic regressions have been estimated to contrast the impact of economic exclusion and income on the probability of being in poor health. Table 7 present estimates of odds ratios (OR) and 95% confidence interval (CI) for fixed effects, as well as the variance of the random intercept and the Median Odds Ratio (MOR) at the household level.

**Table 7:** Multilevel logistic regressions estimates (odds ratios and 95% confidence intervals) and variance components of poor SRH* (n = 1, 588 individuals nested within 975 households)

	Model 0	Model 1	Model 2a	Model 3a	Model 2b	Model 3b
			
			Individual	Income	Household	Income
***Fixed effects***	***Individual level***					

**Age**						
*25-34*		1	1	1	1	1
*35-44*		1.36 [0.71-2.60]	1.42 [0.75-2.71]	1.47 [0.77-2.82]	1.29 [0.67-2.49]	1.36 [0.71-2.62]
*45-54*		1.56 [0.82-2.97]	1.54 [0.82-2.89]	1.69 [0.89-3.21]	1.49 [0.78-2.85]	1.62 [0.85-3.09]
*55-64*		1.31 [0.67-2.59]	1.23 [0.63-2.41]	1.49 [0.75-2.95]	1.18 [0.59-2.33]	1.39 [0.70-2.76]
**Gender**						
*Male*		1.17 [0.80-1.71]	1.50 [1.00-2.22]	1.40 [0.93-2.08]	1.18 [0.80-1.73]	1.17 [0.80-1.71]
*Female*		1	1	1	1	1
**Immigration status**						
*Immigrant*		0.95 [0.54-1.66]	0.78 [0.44-1.38]	0.81 [0.46-1.45]	0.78 [0.44-1.41]	0.85 [0.47-1.51]
*Non immigrant*		1	1	1	1	1
**Marital Status**						
*Single*		1	1	1	1	1
*Couple*		0.83 [0.45-1.52]	0.65 [0.36-1.16]	0.80 [0.44-1.46]	0.84 [0.45-1.57]	0.94 [0.51-1.75]
*Divorced/Separated*		0.54 [0.25-1.15]	0.66 [0.32-1.38]	0.56 [0.26-1.19]	0.57 [0.27-1.22]	0.52 [0.24-1.12]
*Widowed*		1.13 [0.30-4.28]	1.27 [0.34-4.76]	1.26 [0.33-4.74]	1.07 [0.28-4.15]	1.12 [0.29-4.25]
**Education**						
*Less than HS*		1	1	1	1	1
*High school*		1.07 [0.62-1.82]	1.17 [0.68-2.00]	1.19 [0.69-2.03]	1.20 [0.69-2.08]	1.18 [0.68-2.02]
*More than HS*		**0.50 [0.30-0.83]**	**0.58 [0.35-0.97]**	**0.61 [0.36-1.01]**	**0.54 [0.32-0.91]**	**0.56 [0.33-0.93]**
**Employment**						
*Employed*		1	1	1	1	1
*Unemployed*		0.69 [0.23-2.05]	0.67 [0.23-1.96]	0.60 [0.20-1.77]	0.71 [0.24-2.13]	0.65 [0.22-1.94]
*OLF*		**2.26 [1.44-3.57]**	**1.93 [1.21-3.09]**	**1.70 [1.06-2.73]**	**2.28 [1.43-3.63]**	**2.01 [1.27-3.20]**
**Individual Income**			**0.46 [0.32-0.66]**	**0.55 [0.39-0.80]**		

***Fixed effects***	***Household level***					

Household income					**0.49 [0.34-0.70]**	**0.65 [0.45-0.95]**
Index of exclusion		**1.66 [1.35-2.05]**		**1.53 [1.24-1.87]**		**1.51 [1.22-1.88]**

***Random effects***						

Variance (Std. Err.)	1.367 (0.680)	0.640 (0.600)	0.658 (0.580)	0.586 (0.574)	0.824 (0.632)	0.653 (0.603)
MOR	3, 05	2, 14	2, 17	2, 08	2, 38	2, 16

*Adjusted for chronic condition, number of members in the household and province of residence

We observed that self-rated health varied significantly between households as the empty model (M0) indicates a variance of the intercept of 1.367 and an MOR of 3.05. Moreover, the likelihood-ratio test of this empty model indicated the between-household variance was not equal to zero (p = 0.004): this confirmed the need to use multilevel methods rather than a standard logistic regression.

After adding individual characteristics (M1), the variance was reduced to 0.640 and the MOR to 2.14. Respondents with low levels of education were more likely to report being in poor health. Those OLF were more likely to be in poor health than those employed. These effects persisted in subsequent models, remaining significant even if the associations were weakened. However, age, gender, migration status and the province of residence were not significantly associated with health status. The estimates of other variables were unaffected by the presence or absence of the province of residence. This model indicates that the likelihood of being in poor health increased with increasing economic exclusion (OR = 1.66, 95%CI = [1.35-2.05]).

Models 2 introduced income, measured at the individual (M2a) and household (M2b) levels. As with economic exclusion, report of poor health decrease with increasing individual (OR = 0.46, 95%CI = [0.32-0.66]) and household (OR = 0.492, 95%CI = [0.34-0.70]) income.

Finally, when combining both economic exclusion and individual income (M3a) or household income (M3b), effects of all these variables slightly decreased in magnitude but were still statistically significant (OR being respectively 0.55 [0.39-0.80] for individual income and 1.52 [1.24-1.87] for economic exclusion in the first model, and 0.65 [0.45-0.95] household income and 1.51 [1.22-1.87] for economic exclusion in the second one). Here, Model M3a with both individual income and economic exclusion seems to best explain the variation between clusters and offers the best fit for the data.

## Discussion

Contrasting the index of economic exclusion and income significantly contributes to our understanding of social inequalities in health in Canada. Our analyses indeed show that, while economic exclusion is inversely correlated with both individual and household income, these are not perfectly overlapping constructs. Indeed, not only are these indicators weakly correlated, but they also point to slightly different sociodemographic groups at risk of low income and economic exclusion. Moreover, the respective associations with health are of comparable significance, but when these income and economic exclusion indicators are included together in the same model, they point to independent and cumulative, not redundant effects. In sum, economic exclusion index measures additional aspects that are not completely addressed with income, such as the effective hardship or level of economic 'well-being'. These findings also confirm the validity of the tools and the index in the CPHS-pilot.

Previous work done at Statistics Canada [38] shows that items as well as the level of analysis (household or individual) may differ between countries. However, using a ratio of the number of items experienced to the total number of questions, we were able to create comparable information despite survey and country idiosyncrasies. We argue that this is a fruitful strategy first because it allows us to keep respondents with missing information within the present study (only 23 household with missing information on all items were not considered), and second, because it improves the comparability with other international panel surveys for future analyses.

These results reinforce previous literature suggesting that economic exclusion does not exactly measure the same concept as income does [17]. Yet, while the positive impact of income (both individual and household) on health has been extensively described in the literature, publication on the impact of material deprivation or economic exclusion on health are scant. Previous studies did find that wealth has a positive effect on health [40-43], while material deprivation [20-25] and economic hardship [26-31] have negative effect on self rated health. However, the operationalisation of material deprivation often consisted of income and employment status, or, at best, household goods. Thus, these effects conflate the impact of income with more explicit measures of economic exclusion. We found only few studies comparing income and other measures of economic position in the literature and all found that the impact on health status of (monetary) wealth [40] or economic hardship using a single indicator [30] differed than the one from income. Our study goes further using a more complex and multidimensional index of economic exclusion that is easily replicable in other surveys.

In terms of limitations, we ought to restate that the CHPS-pilot is not a nationally representative survey. Moreover, it is cross-sectional, which restricts the causal implications of our research. Some studies analyzing longitudinal data have shown a dose response relationship between poverty trajectories and health, those persistently poor having worse health outcomes than the non-poor or transient [29,30,44]. However, one advantage of our data is that the income information is taken directly from the tax records for a substantial proportion of our sample. Therefore, while the index of economic exclusion may be proned to self-reported bias, it is unlikely that the income tax report for the previous financial year suffers from the same limitations. Of course, this temporal lag may also explain the weak correlation between income and economic exclusion: indeed, it is possible that the latter reflects more current circumstances that did not prevail a year ago. This possibility points of course to the need for longitudinal data examining these processes in a dynamic way. Another explanation that could explain the weak correlation (but not the cumulative effect) between income and economic exclusion is that we had only access to income information before tax, and not after tax. This is a limitation (common to many surveys) in that it does indeed not accurately reflect disposable income. Finally, these processes should be examined not only with income, but also with low income cut-off measures to get closer to the estimation of poverty status.

Objective measures such as income have intuitive appeal. However, we would argue that income is only a rough indicator of lack of economic resources, which limits its usefulness for public policy. Even though concepts such as wealth, material deprivation or economic hardship might better assess this lack of economic resources, it seems crucial to take into account the source of stress stemming from perceived inadequacy of one's available economic resources [45,46]. Although income is associated with adverse events, this association is often mediated by variety of psychological, social and environmental factors [26,47,48]. There is therefore a growing interest to look at more complex and extend concepts and social factors to understand and explain health inequalities. The Commission on Social Determinants of Health and more specifically its Social Exclusion Knowledge Network recently defined a list of social determinants leading to health inequalities [4,49]. Among them, they highlighted social exclusion and developed a parsimonious and comprehensive framework composed of four dimensions: economic, social, political and cultural. However, much remains to be done to operationalise these four dimensions adequately and comparably between surveys and countries. One of the main strengths of this framework is its flexibility for identifying exclusion in one dimension (e.g. economically excluded) but maybe not in another (e.g. socially included). Thus, it proposes that it is the sum of these dimensions that leads to greater levels of social exclusion. Another advantage is the depiction of inclusion to exclusion as a continuum rather than a dichotomous scale. This work highlights the need and the importance of measuring multiple dimensions of economic exclusion in panel surveys, and suggests that we might learn even more by examining the four dimensions of social exclusion proposed by the Social Exclusion Knowledge Network. Indeed, the extension of the measures available in the CHPS-Pilot to the four dimensions of economic, cultural, social and political exclusion could highlight fruitful avenues for future public health, social and intersectorial policies.

## Conclusion

The contribution of this paper was to explicitly distinguish, both conceptually and empirically, between income and economic exclusion, one of the main dimensions of social exclusion. Thus, we show that, in the Canadian context, different groups can be at risk of lower income and of economic exclusion. Moreover, while an inverse correlation of economic exclusion with income exists, it is weak, and suggests that these variables measure different concepts. The independence of these two measures demonstrates construct validity of our index.

For instance, associations differed by gender and age. While men tended to report both higher individual income, there was no significant gender difference in the index of economic exclusion. This difference might highlight the increasing empowerment of women in Canada despite persistent income discrepancies. While income increases up to a certain age and then decreases, economic exclusion appeared to linearly decrease with increasing age, probably as the result of accumulation of goods. Thus, even though they report lower incomes, women and people over 55 report a good level of economic integration. These differences may be also due to the positive impact of social transfers and social policies more generally in Canada. Social policies could help people with low income face economic hardship in their daily life. As an additional example, those who reported a chronic condition had lower incomes, but did not appear to face significantly higher economic exclusion. As argued by Gannon et al. [50], this could be a consequence of accessibility policies favoring the inclusion of disabled individuals.

However, despite these comforting findings, we did also identify groups that were vulnerable to both lower income and economic exclusion, and already known to be at risk of social exclusion [51,52]: those with poor self-rated health (though not necessarily - yet- diagnosed with a chronic condition), the unemployed and immigrants. This observation highlights the need for a better understanding of the interplay between income, social policies and the risk of economic exclusion.

## List of abbreviations

CHPS-Pilot: Canadian Household Panel Survey; FHR: Food, housing and financial risk or economic hardship; FP: Financial products or wealth; MOR: Median odds ratio; MTD: Material deprivation; OLF: Out of the labour force; OR: Odds ratios; 95%CI: 95% confidence interval.

## Competing interests

The authors declare that they have no competing interests.

## Authors' contributions

ER, BAL, MK and AQV have made contributions to analysis and interpretation of data. All authors have been involved in drafting or revising the manuscript, read and approved the final manuscript.

## References

[B1] Buckley NJ Denton FT Robb AL Spencer BG Socio-economic influences on the health of older Canadians: Estimates based on two longitudinal surveys Can Public Policy-Anal Polit 2006 32 59 83 10.2307/3552243

[B2] Trovato F Heyen NB A varied pattern of change of the sex differential in survival in the G7 countries J Biosoc Sci 2006 38 391 401 10.1017/S0021932005007212 16613623

[B3] Trovato F Lalu N From divergence to convergence: The sex differential in life expectancy in Canada, 1971-2000 Can Rev Sociol Anthropol-Rev Can Sociol Anthrol 2007 44 101 122 10.1111/j.1755-618x.2007.tb01149.x 17644998

[B4] CSDH Closing the gap in a generation: health equity through action on the social determinants of health. Final Report of the Commission on Social Determinants of Health 2008 Geneva: World Health Organization 10.1016/S0140-6736(08)61690-6 18994664

[B5] Mercier E Mendell A An Act to Combat Poverty and Social Exclusion (R.S.Q., Chapter L-7). History Briefing Note. For up-to-date knowledge relating to healthy public policy 2009 Quebec: National Collaborating Centre for Healthy Public Policy

[B6] Mendell A Comprehensive policies to combat poverty across Canada, by Province. Preliminary document for discussion 2009 National Collaborating Centre for Healthy Public Policy

[B7] Bartley M Health inequality: an introduction to theories, concepts and methods 2004 Oxford: Polity Press

[B8] Braveman PA Cubbin C Egerter S Chideya S Marchi KS Metzler M Posner S Socioeconomic status in health research - One size does not fit all JAMA-J Am Med Assoc 2005 294 2879 2888 10.1001/jama.294.22.2879 16352796

[B9] Macintyre S McKay L Der G Hiscock R Socio-economic position and health: what you observe depends on how you measure it J Public Health 2003 25 288 294 10.1093/pubmed/fdg089 14747587

[B10] Turrell G Income non-reporting: implications for health inequalities research J Epidemiol Community Health 2000 54 207 214 10.1136/jech.54.3.207 10746115 PMC1731636

[B11] Henly JR Danziger SK Offer S The Contribution of Social Support to the Material Well-Being of Low-Income Families Journal of Marriage and Family 2005 67 122 140 10.1111/j.0022-2445.2005.00010.x

[B12] Krause N Anticipated support, received support, and economic stress among older adults Journals of Gerontology - Series B Psychological Sciences and Social Sciences 1997 52 P284 P293 10.1093/geronb/52b.6.p284 9403517

[B13] Whelan CT Layte R Maitre B Nolan B Income, deprivation, and economic strain. An analysis of the European community household panel European Sociological Review 2001 17 357 372 10.1093/esr/17.4.357

[B14] Rawls J A theory of justice 1971 Cambridge: Harvard University Press and Clarendon Press

[B15] Sen A Poor, relatively speaking Oxford Economic Papers-New Series 1983 35 153 169

[B16] Berthoud R Bryan M Bardasi E The dynamics of deprivation: the relationship between income and material deprivation over time Families and Children Strategic Analysis Programme 2004 Colegate: University of Essex 126 vol. Research Report No 219

[B17] Nolan B Whelan CT Resources, Deprivation and Poverty 1996 Oxford: Clarendon Press

[B18] Ringen S Direct and indirect measure of poverty Journal of Social Policy 1988 17 351 365 10.1017/S0047279400016858

[B19] Tsakloglou P Papadopoulos F Poverty, material deprivation and multi-dimensional disadvantage during four life stages: Evidence from the ECHP 2001 Athens: Aldershot and CHeltenham

[B20] Baker D Taylor H The relationship between condition-specific morbidity, social support and material deprivation in pregnancy and early motherhood Soc Sci Med 1997 45 1325 1336 10.1016/S0277-9536(97)00059-2 9351152

[B21] Baumann M Spitz E Guillemin F Ravaud JF Choquet M Falissard B Chau N Lorhandicap G Associations of social and material deprivation with tobacco, alcohol, and psychotropic drug use, and gender: a population-based study International Journal of Health Geographics 2007 6 50 10.1186/1476-072X-6-50 17996098 PMC2211297

[B22] Benzeval M Judge K Smaje C Beyond class, race, and ethnicity. Deprivation and health in Britain Health Services Research 1995 30 163 177 7721590 PMC1070047

[B23] Bobak M Pikhart H Rose R Hertzman C Marmot M Socioeconomic factors, material inequalities, and perceived control in self-rated health: cross-sectional data from seven post-communist countries Soc Sci Med 2000 51 1343 1350 10.1016/S0277-9536(00)00096-4 11037221

[B24] Borrell C Muntaner C Sola J Artazcoz L Puigpinos R Benach J Noh S Immigration and self-reported health status by social class and gender: the importance of material deprivation, work organisation and household labour J Epidemiol Community Health 2008 62 671 675 10.1136/jech.2006.055269 18431832

[B25] Groffen DAI Bosma H van den Akker M Kempen G van Eijk JTM Material deprivation and health-related dysfunction in older Dutch people: findings from the SMILE study European Journal of Public Health 2008 18 258 263 10.1093/eurpub/ckm119 18160391

[B26] Brown GW Moran PM Single mothers, poverty and depression Psychological Medicine 1997 27 21 33 10.1017/S0033291796004060 9122302

[B27] Butterworth P Rodgers B Windsor TD Financial hardship, socio-economic position and depression: Results from the PATH Through Life Survey Social Science & Medicine 2009 69 2 229 237 10.1016/j.socscimed.2009.05.008 19501441

[B28] Mirowsky J Ross CE Age and the effect of economic hardship on depression Journal of health and social behavior 2001 42 132 150 10.2307/3090174 11467249

[B29] Lynch JW Kaplan GA Shema SJ Cumulative impact of sustained economic hardship on physical, cognitive, psychological, and social functioning New England Journal of Medicine 1997 337 1889 1895 10.1056/NEJM199712253372606 9407157

[B30] Ahnquist J Fredlund P Wamala SP Is cumulative exposure to economic hardships more hazardous to women's health than men's? A 16-year follow-up study of the Swedish Survey of Living Conditions J Epidemiol Community Health 2007 61 331 336 10.1136/jech.2006.049395 17372294 PMC2652943

[B31] Angel RJ Frisco M Angel JL Chiriboga DA Financial strain and health among elderly Mexican-origin individuals Journal of Health and Social Behavior 2003 44 536 551 10.2307/1519798 15038148

[B32] Heisz A Results from the Canadian Household Panel Survey Pilot. Statistics Canada 2011 http://www.statcan.gc.ca/pub/89-648-x/89-648-x2011001-eng.htm

[B33] Berthoud R Blekesaune M Hancock R Ageing, income and living standards: evidence from the British Household Panel Survey Ageing Soc 2009 29 1105 1122 10.1017/S0144686X09008605

[B34] Jones AM Wildman J Health, income and relative deprivation: Evidence from the BHPS J Health Econ 2008 27 308 324 10.1016/j.jhealeco.2007.05.007 18207266

[B35] Pevalin DJ Socio-economic inequalities in health and service utilization in the London Borough of Newham Public Health 2007 121 596 602 10.1016/j.puhe.2006.12.015 17499320

[B36] Pevalin DJ Taylor MP Todd J The dynamics of unhealthy housing in the UK: A panel data analysis Hous Stud 2008 23 679 695 10.1080/02673030802253848

[B37] Torsheim T Currie C Boyce W Kalnins I Overpeck M Haugland S Material deprivation and self-rated health: a multilevel study of adolescents from 22 European and North American countries Soc Sci Med 2004 59 1 12 10.1016/j.socscimed.2003.09.032 15087138

[B38] Heisz A Langevin M Fréchet G, Gauvreau D, Poirier J Material deprivation in household panel surveys: International evidence and lessons for Canada Statistiques Sociales, pauvreté et exclusion sociale: hommage à Paul Bernard 2011 Les Presses de l'Université de Montréal 269 27712120857

[B39] Larsen K Merlot J Appropriate assessment of neighborhood effects on individual health: Integrating random and fixed effects in multilevel logistic regression Am J Epidemiol 2005 161 81 88 10.1093/aje/kwi017 15615918

[B40] Aittomaki A Martikainen P Laaksonen M Lahelma E Rahkonen O The associations of household wealth and income with self-rated health - A study on economic advantage in middle-aged Finnish men and women Soc Sci Med 2010 71 1018 1026 10.1016/j.socscimed.2010.05.040 20598791

[B41] Avendano M Glymour MM Life course inheritances, wealth and health: Examining causal effects in 11 European countries. American Journal of Epidemiology 2010 171 S16 S16

[B42] Hairi FM Mackenbach JP Andersen-Ranberg K Avendano M Does socio- economic status predict grip strength in older Europeans? Results from the SHARE study in non- institutionalised men and women aged 50+ J Epidemiol Community Health 2010 64 829 837 10.1136/jech.2009.088476 19884112

[B43] Hajat A Kaufman JS Rose KM Siddiqi A Thomas JC Do the wealthy have a health advantage? Cardiovascular disease risk factors and wealth Soc Sci Med 2010 71 1935 1942 10.1016/j.socscimed.2010.09.027 20970902 PMC12081007

[B44] Duncan GJ Brooks-Gunn J Klebanov PK Economic deprivation and early childhood development Child development 1994 65 2 Spec No 296 318 7516849

[B45] Hazelrigg LE Hardy MA Perceived income adequacy among older adults: Issues of conceptualization and measurement, with an analysis of data Research on Aging 1997 19 69 107 10.1177/0164027597191004

[B46] Mirowsky J Ross CE Economic hardship across the life course American Sociological Review 1999 64 548 569 10.2307/2657255

[B47] Felner RD Brand S DuBois DL Adan AM Mulhall PF Evans EG Socioeconomic disadvantage, proximal environmental experiences, and socioemotional and academic adjustment in early adolescence: investigation of a mediated effects model Child development 1995 66 774 792 10.2307/1131950 7789201

[B48] McLeod JD Kessler RC Socioeconomic status differences in vulnerability to undesirable life events Journal of health and social behavior 1990 31 162 172 10.2307/2137170 2102495

[B49] SEKN Understanding and tackling social exclusion. Final Report of the Social Exclusion Knowledge Network of the Commission on Social Determinants of Health 2008 Geneva: World Health Organization

[B50] Gannon B Nolan B The impact of disability transitions on social inclusion Soc Sci Med 2007 64 1425 1437 10.1016/j.socscimed.2006.11.021 17194515

[B51] Shaw M Dorling D Smith GD Marmot M, Wilkinson RG Poverty, Social Exclusion and Minorities Social Determinants of Health 1999 Oxford: Oxford University Press 211 239

[B52] Galabuzi G-E Dennis Raphael. Toronto, ON Social exclusion Social determinants of health. Canadian perspectives 2004 Canadian's Scholars' Press Inc. 235 252

